# Systemically administered collagen‐targeted gold nanoparticles bind to arterial injury following vascular interventions

**DOI:** 10.14814/phy2.13128

**Published:** 2017-02-27

**Authors:** Molly Wasserman Meyers, Jonathan S. Rink, Qun Jiang, Megan E. Kelly, Janet M. Vercammen, Colby S. Thaxton, Melina R. Kibbe

**Affiliations:** ^1^ Department of SurgeryNorthwestern UniversityChicagoIllinois; ^2^ Simpson Querrey Institute for BioNanotechnologyChicagoIllinois; ^3^ Department of UrologyNorthwestern UniversityChicagoIllinois; ^4^ Department of SurgeryUniversity of North CarolinaChapel HillNorth Carolina; ^5^ Present address: Department of SurgeryUniversity of North Carolina at Chapel HillChapel HillNorth Carolina

**Keywords:** Atherosclerosis, neointimal hyperplasia, restenosis, vascular biology, vascular disease

## Abstract

Surgical and endovascular therapies for severe atherosclerosis often fail due to the development of neointimal hyperplasia and arterial restenosis. Our objective was to synthesize, characterize, and evaluate the targeting specificity and biocompatibility of a novel systemically injected nanoparticle. We hypothesize that surface‐functionalization of gold nanoparticles (AuNPs) with a collagen‐targeting peptide will be biocompatible and target specifically to vascular injury. 13 nm AuNPs were surface functionalized with a peptide‐molecular fluorophore and targeted to collagen (T‐AuNP) or a scrambled peptide sequence (S‐AuNP). After rat carotid artery balloon injury and systemic injection of T‐AuNP or S‐AuNP, arteries and organs were harvested and assessed for binding specificity and biocompatibility. The T‐AuNP bound with specificity to vascular injury for a minimum of 24 h. No significant inflammation was evident locally at arterial injury or systemically in major organs. The T‐AuNP did not impact endothelial cell viability or induce apoptosis at the site of injury in vivo. No major changes were evident in hepatic or renal blood chemistry profiles. Herein, we synthesized a biocompatible nanoparticle that targets to vascular injury following systemic administration. These studies demonstrate proof‐of‐principle and serve as the foundation for further T‐AuNP optimization to realize systemic, targeted delivery of therapeutics to the sites of vascular injury.

## Introduction

Atherosclerosis is the leading cause of death and disability in the United States, affecting more than one in three Americans (Mozaffarian et al. 2015). With an aging population, the prevalence of coronary artery disease (CAD) and peripheral arterial disease (PAD) is anticipated to increase. Current surgical and endovascular therapies to treat severe CAD and PAD, including balloon angioplasty and stenting, endarterectomy, and surgical bypass grafting, are costly, and often fail as a result of neointimal hyperplasia and subsequent arterial restenosis (Clowes et al. 1983; Groschel et al. 2005; Inoue et al. 2011; Kornowski et al. 1998; Welt and Rogers 2002). Drug‐eluting stents, designed to provide arterial patency while preventing neointimal hyperplasia, indiscriminately inhibit cell proliferation within the stented artery, causing delayed reendothelialization and resulting in an unacceptable rate of in‐stent thrombosis and death (Inoue et al. 2011; Kastrati et al. 2005). In addition, patients who undergo arterial angioplasty with stenting require long‐term dual antiplatelet therapy, placing them at increased risk of bleeding complications (Bhatt et al. 2006; Steinhubl et al. 2002). There exists a clear need for new technology that will enable safe revascularization and improve atherosclerotic arterial patency while minimizing patient exposure to long‐term risks.

Nanotechnology is an emerging platform for the targeted delivery of therapeutics and imaging modalities (Agyare and Kandimalla 2014; Morgan et al. 2016; Rink et al. 2013; Thaxton et al. 2016). Critical to the design of nanoparticle therapeutics is the ability to integrate multiple functions into complex nanosystems, such as targeting moieties, therapeutic payloads and/or imaging modalities, while remaining biocompatible. Nanobiomedicine is being increasingly popularized in the cancer literature (Davis et al. 2010; Hrkach et al. 2012; Tabernero et al. 2013), whereas few targeted nanotherapeutics exist in the vascular arena. An interesting approach is to design a nanotherapeutic that can be systemically delivered but targeted specifically to the site of vascular injury. This “smart technology” delivery platform has the advantages of a minimally invasive systemic intravascular route of administration, the opportunity for repeat dosing, targeted delivery of a therapeutic agent to the site of interest, limited systemic toxicity or off‐target side effects due to its targeting capabilities, and avoidance of indwelling prosthetic devices (Gupta 2011).

Our initial objective was to design and characterize a biocompatible nanoconstruct that specifically targets the site of vascular injury. Following vascular interventions, the endothelial cell lining is disrupted, exposing the collagen IV matrix below. Targeting a nanoconstruct to collagen IV should direct the nanoparticles to the site of vascular injury while bypassing healthy vasculature. We demonstrate here that systemic administration of a collagen IV‐targeted gold nanoparticle (AuNP) binds with specificity to arterial injury and is biocompatible with cells of the vascular wall. This research lays the foundation for the development of a systemically administered, targeted nanoparticle to treat vascular disease.

## Materials and Methods

### Synthesis of the collagen‐targeted (T) or scrambled (S) gold nanoparticle (AuNP)

**Figure 1:**
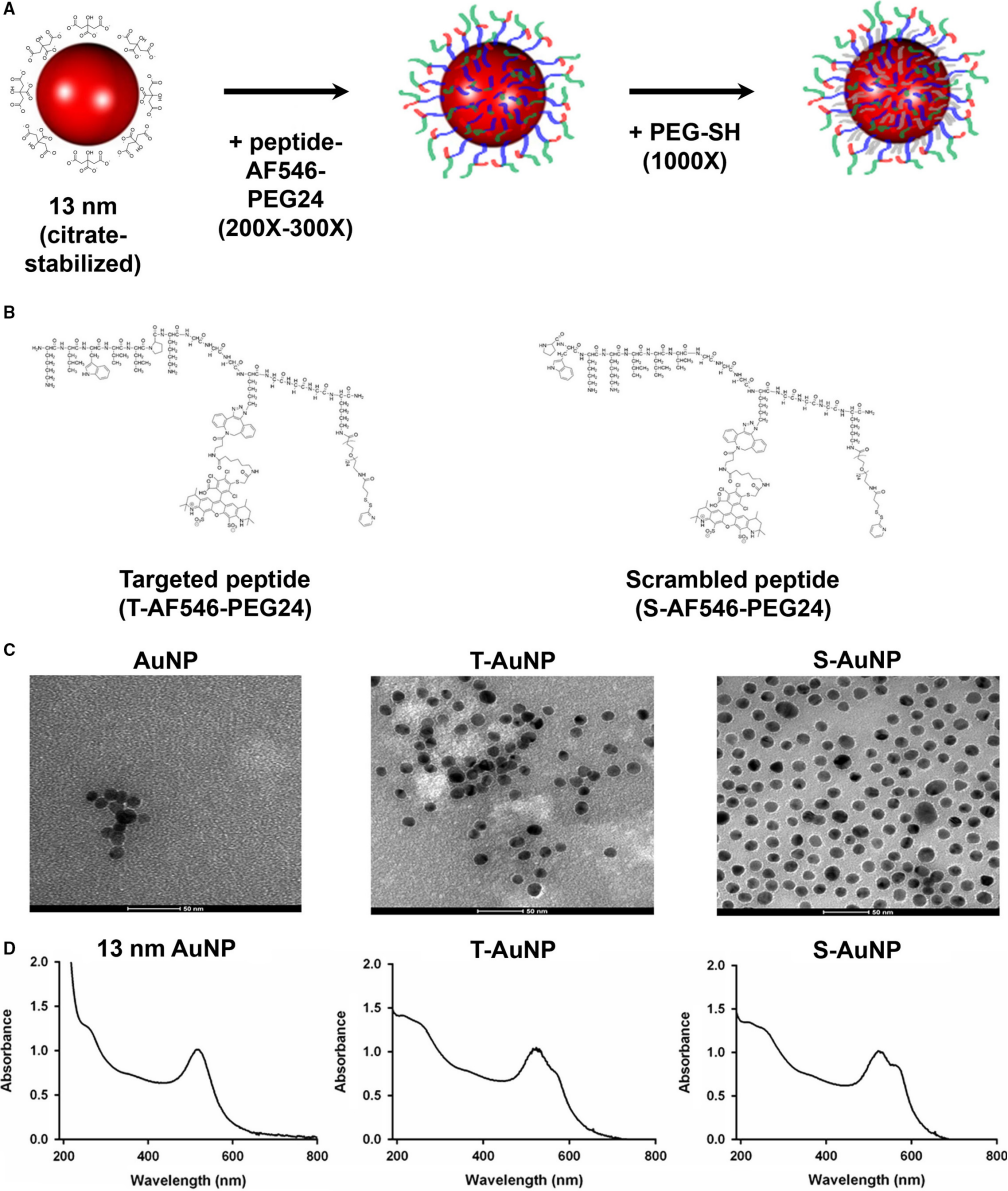
Synthesis and characterization of the T‐AuNP and S‐AuNP. (A) Synthesis scheme of the T‐AuNP and S‐AuNP. Citrate‐stabilized 13 nm AuNPs were surface functionalized with either the targeted peptide‐molecular fluorophore‐PEG‐thiol or scrambled peptide‐molecular fluorophore‐PEG‐thiol molecules, followed by PEG‐SH (MW = 1000) to fill any remaining unfunctionalized spaces. (B) Chemical structure of the targeted (CBP‐AF546‐PEG24‐SH; left) and scrambled (SCR‐AF546‐PEG24‐SH; right) peptide‐fluorophore‐PEG‐thiol molecules. (C) Transmission electron microscopy (TEM) imaging of nanoparticle constructs. TEM images of 13 nm AuNP (left), T‐AuNP (center) and S‐AuNP (right) taken at 150,000 × magnification. Scale bar = 50 nm. (D) UV/Vis spectra of 13 nm AuNP (left), T‐AuNP (center) and S‐AuNP (right). TEM, Transmission electron microscopy.

The collagen‐targeted and scrambled peptide conjugates were synthesized by the Peptide Synthesis Core at Northwestern University. The peptide conjugates consist of a targeted or scrambled sequence (collagen targeted: H2N‐KLWVLPK‐COOH; scrambled: H2N‐PWKKLLV‐COOH) and a modified lysine residue containing the fluorescent dye Alexa Fluor 546 (AF546) (Thermo Fisher Scientific, Waltham, MA, #A20002), conjugated to a 24‐unit polyethylene glycol (PEG24) containing a terminal disulfide group (Fig. 1A and B). The peptide and fluorophore are separated from the PEG24 by GGG spacers. The final peptide was purified by high‐performance liquid chromatography (HPLC).

Citrate‐stabilized, 13 nm diameter gold nanoparticles were synthesized as previously described (Rosi et al. 2006). The size and stability of the AuNPs were measured by dynamic light scattering (DLS) and UV‐Vis spectroscopy, respectively. For surface functionalization with the targeted or scrambled peptide containing ligands, AuNPs (10 nmol/L) were incubated with a 200‐fold molar excess of collagen‐targeted or a 300‐fold molar excess of scrambled peptide conjugates for 1 h at room temperature. Next, a PEG‐SH (MW = 1000) molecule was added to the solution in 1000‐fold molar excess with respect to the AuNP and allowed to incubate overnight at room temperature to passivate unreacted regions of the AuNPs (Fig. 1A). The molar excess values for the collagen‐targeted and scrambled molecules was empirically determined for both maximal ligand loading as well as equivalent loading of each molecule. After overnight incubation, the AuNP conjugates were purified by centrifugation (15,900 RCF for 30 min). After removal of the supernatant, the resulting oily pellet was washed once with sterile water and the nanoparticles purified again by centrifugation. Resulting T‐AuNP and S‐AuNP concentrations were determined using UV‐Vis spectroscopy and Beer's law, A=Ɛ*l*c, where A is the absorbance of the nanoparticles at ~520 nm, Ɛ is the extinction coefficient for 13 nm AuNPs (2.4 *10^8^ M^−1 ^cm^−1^), l is the path length (1 cm), and c is the concentration of the AuNP solution.

### Characterization of T‐AuNP and S‐AuNP

T‐AuNPs and S‐AuNPs were characterized with respect to size (DLS, nm), surface charge (zeta potential, mV), stability (*λ*_max_), and loading of the fluorescent ligands. For DLS measurements, 1 nmol/L T‐AuNP or S‐AuNP was analyzed using the Malvern Zetasizer (Malvern Instruments Ltd, Worcestershire, UK). Data are presented as mean hydrodynamic diameter ± standard deviation. The surface charge of the T‐AuNP and S‐AuNP (concentration: 1 nmol/L) was quantified using the Malvern Zetasizer (Malvern Instruments Ltd). Data are presented as mean zeta potential ± standard deviation. Stability of the T‐AuNP and S‐AuNP was measured using UV‐Vis spectroscopy with reference made to citrate‐stabilized AuNPs (*λ*_max_ = 520 nm). Peptide loading onto the AuNPs was quantified using a fluorescent microplate reader whereby surface ligands labeled with a molecular fluorophore were liberated from the AuNPs using potassium cyanide to dissolve the AuNP core. After digestion, the fluorescence was then read at 565 nm on the BioTek Synergy 2 plate reader (BioTek, Winooski, VT) and the number of Alexa Fluor 546 molecules per nanoparticle was quantified using a standard curve of each peptide‐AF546‐PEG ligand. Data are presented as the mean number of ligands per nanoparticle ± standard deviation.

### Transmission electron microscopy

Forceps were used to hold transmission electron microscopy (TEM) grids (200 mesh copper grids covered with carbon film; Electron Microscopy Sciences, Hatfield, PA) in place while a 10 *μ*L drop of 5 nmol/L 13 nm AuNP, T‐AuNP or S‐AuNP was added. The solution was allowed to adsorb onto the grid for 10 min, with the excess solution wicked away using filter paper. The grids were counterstained with 10 *μ*L of uranyl acetate (2% w/w) for 10 min, and the excess solution wicked away using filter paper. The grids were allowed an additional 15 min to air dry prior to storage in a dessicator until TEM imaging was performed, using a FEI Tecnai Spirit G2 (FEI, Hillsboro, OR) operating at 120 kV. Images were taken at 150,000 × magnification for all samples.

### Animal surgery

All animal procedures were performed in accordance with the principles outlined in the Guide for the Care and Use of Laboratory Animals, published by the National Institutes of Health (NIH Publication 85–23, 1996), and approved by the Northwestern University Animal Care and Use Committee. Adult male Sprague–Dawley rats (Harlan, Indianapolis, IN), 10‐weeks‐old, weighing 350–450 g underwent the rat carotid artery balloon injury model as previously described (Vavra et al. 2011). After dissection and isolation of the common carotid artery and ligation of the external carotid artery, heparin (80 U/kg) was administered via tail vein injection. The internal and common carotid arteries were then clamped; uniform injury was created in the common carotid artery by inflation of a No. 2 French arterial embolectomy catheter (Edwards Lifesciences, Irvine, CA) inserted into the external carotid artery to 5 atmospheres of pressure for 5 min. After angioplasty and catheter removal, the external carotid arteriotomy site was ligated and 0.7, 1.4, 2.0, or 2.7 mg peptide‐fluorophore‐PEG‐SH conjugate/kg rat body weight of the T‐AuNP (*N* = 29) or S‐AuNP (*N* = 10) was injected into the inferior vena cava (IVC) superior to the renal veins. For the sake of consistency within this study and to reduce variabiltiy, we used the IVC as the site for injection. However, for subsequent studies, we have used tail vein injection and achieved similar results. Immediately after injection, the arterial clamps on the internal and common carotid arteries were removed and flow was restored. Rats were killed at 20 min (T‐AuNP, *N* = 14, S‐AuNP, *N* = 7), 2 h (T‐AuNP, *N* = 3), 24 h (T‐AuNP, *N* = 3, S‐AuNP, *N* = 3), 48 h (T‐AuNP, *N* = 3), 72 h (T‐AuNP, *N* = 3), and 96 h (T‐AuNP, *N* = 3). Additional control rats underwent balloon injury without systemic nanoparticle injection and were killed at 24 h (*N* = 3).

### Tissue processing

Bilateral carotid arteries, liver, lung, spleen, and kidneys were harvested following in‐situ perfusion with 1× phosphate‐buffered solution (PBS, 400 mL) followed by a 2% paraformaldehyde bath for 1 h at 4°C. Tissue was cryoprotected in a 30% sucrose bath for 1 h at 4°C. Vessels and organs were coated with Optimum Cutting Temperature O.C.T.^™^ compound (Tissue Tek, Hatfield, PA) and flash‐frozen in liquid nitrogen. Cryosections of 5 *μ*m were then cut at equally spaced intervals along the length of the artery or organ using a ThermoScientific CryoStar NX70 cryostat (Thermo Fisher Scientific, Waltham, MA).

### Morphometric analysis

Carotid arteries and organs were examined histologically at 24 h for evidence of architectural changes using routine hematoxylin–eosin (H&E) staining. Six evenly spaced sections per artery and organ were morphometrically analyzed at the 5×, 10×, and 25× objectives using an Olympus BH2 microscope (Olympus, Tokyo, Japan).

### Immunofluorescence analysis

Bilateral carotid arteries and organs were analyzed for T‐AuNP and S‐AuNP binding using high‐resolution digital image microscopy with a Zeiss AxioCam HRm microscope (Zeiss, Oberkochen, Germany) and AxioVision Rel 4.8 software (Zeiss) at 5×, 10×, and 25× objectives. The Green Fluorescent Protein (GFP) filter set 38 (Zeiss) was used to visualize the arterial elastic lamina, and the Cy3 filter set 43 (Zeiss), using excitation and emission wavelengths of 550–575 nm and 605–670 nm, respectively, was used to visualize the fluorophore tag AF546 (Thermo Fisher Scientific, Waltham, MA, #A20002).

Arteries and organs were examined for evidence of neutrophil or monocyte/macrophage inflammation using immunofluorescence staining. Arteries were also assessed for the presence of endothelial cells using immunofluorescent staining for CD31. Sections were fixed with 2% paraformaldehyde for 20 min at 4°C, then permeabilized with 0.3% Triton‐X100 for 10 min, followed by a 2 min rinse with 1× PBS. Primary antibody in IHC‐Tek (IHC World, Ellicott City, MD) diluent (200 *μ*L) was applied at room temperature for 1 h: myeloperoxidase (neutrophil, 1:100; Abcam, Cambridge, UK, #ab9535), anti‐ED1 (monocyte/macrophage, 1:1000; Serotec, Raleigh, NC, #MCA341R), anti‐CD31 (endothelial cells, 1:500; Novus, St. Louis, MO, #NB100‐2284). Secondary antibody in 1× PBS (200 *μ*L) was applied at room temperature for 1 h: myeloperoxidase (goat anti‐rabbit IgG Alexa Fluor 555, 1:100; Invitrogen, #A‐21428), ED1 (goat anti‐mouse IgG Alexa Fluor 555, 1:100; Invitrogen, #A‐21422), CD31 (goat anti‐rabbit IgG Alexa Fluor 555, 1:100; Invitrogen, #A‐21428). Nuclei were stained with DAPI (Invitrogen) in 1× PBS (1:500) for 5 min at room temperature. Coverslips were affixed with ProLong Gold Antifade Reagent (Invitrogen) and slides were light protected and allowed to dry overnight.

Slides were then analyzed for the presence of neutrophils, macrophages, and endothelial cells using high‐resolution digital imaging microscopy with a Zeiss AxioCam HRm microscope (Zeiss) and AxioVision Rel 4.8 software (Zeiss) at 5×, 10×, and 25× objectives. The Green Fluorescent Protein (GFP) filter set 38 (Zeiss) was used to visualize the elastic lamina, the DAPI filter set 49 (Zeiss) was used to visualize the DAPI‐stained nuclei, and the Cy5 filter set 50 (Zeiss) was used to visualize the secondary antibody stain Alexa Fluor 555 for neutrophils, macrophages, and endothelial cells. Fluorescent pixels of the arterial cross sections were quantified in high power field images (20×) using Image J Software.

### TUNEL staining

The DeadEnd Colorimetric terminal deoxynucleotidyl transferase dUTP nick end labeling (TUNEL) System (Promega, Madison, WI, #G7130) assay was used to assess for apoptotic endothelial cells on arterial cross sections 24 h after arterial injury and exposure to the T‐AuNP and S‐AuNP. Sections were analyzed at the 40× objective using an Olympus BH2 microscope (Olympus, Tokyo, Japan).

### Rigor and reproducibility

Immunofluorescent staining (CD31, MPO, ED1) was performed by two individuals, both blinded to treatment group. High‐resolution digital image fluorescent microscopy was performed by a single nonblinded individual to aid in imaging consistency and standardization across all specimens. All animal surgeries were performed by a single individual to preserve consistency in surgical technique. Tissue processing was performed by three separate individuals, all blinded to treatment group. Morphometric analyses were performed by two individuals, both blinded to treatment group.

### Cell culture

Rat aortic endothelial cells (RAEC), rat aortic smooth muscle cells (RASMC), and rat aortic adventitial fibroblasts (RAAF) were obtained from Cell Applications, Inc. (San Diego, CA; Lot #2725). RAEC were maintained in Rat Endothelial Cell Growth Media (Cell Applications, #R211‐500), and RASMC and RAAF were maintained in complete medium containing 50:50 Dulbecco's Modified Eagle's Medium – Low Glucose) and Ham's F12 (Invitrogen #11885‐092) supplemented with 10% fetal bovine serum (FBS, Invitrogen, #10082147), 100 U/mL penicillin (Invitrogen, #15140122), 100 *μ*g/mL streptomycin (Invitrogen, #15140122), and 4 mmol/L _l_‐glutamine (VWR, West Chester, PA, #45000‐676). All cells were incubated at 37°C in 95% air and 5% CO_2_. For all RAEC experiments, plate wells were pretreated with Attachment Factor Solution (Cell Applications#123‐500) for 3 h at 37°C. For all experiments, cells used were between passages three and eight.

### Cell binding/internalization

RAEC, RASMC, and RAAF were plated onto 96‐well black glass bottom plates at a density of 1.0 × 10^4^ cells/well. After 24 h, cells were treated with a 45 nmol/L concentration of the T‐AuNP or S‐AuNP for a total volume of 100 *μ*L/well (*N* = 16 wells/control group/time point, *N* = 32 wells/T‐AuNP treatment group/time point, *N* = 16 wells/S‐AuNP treatment group/time point; experiment was repeated twice). Cells were exposed to the treatment for 4 h or 24 h, after which wells were rinsed, fixed in 4% paraformaldehyde, and imaged with the Nikon A1R+ (B) GaAsP Confocal Laser Microscope System (Nikon, Tokyo, Japan).

### Cell proliferation, viability, and death

RAEC, RASMC, and RAAF were plated onto 12‐well plates at a density of 1.0 × 10^5^ cells/well. After 24 h, cells were treated with a 45 nmol/L concentration of the T‐AuNP or S‐AuNP for a total volume of 1000 *μ*L/well (*N* = 3 wells/treatment group/time point; experiment repeated 4 times). Trypan blue (Sigma Aldrich, St. Louis, MO, #T8154) staining was performed at 4 h and 24 h to assess for cell viability, death, and proliferation, as previously published (Louis and Siegel 2011).

### Statistical analysis

All results are provided as mean ± the standard error of the mean. SigmaPlot (Systat Software, Inc., Chicago, IL) was used to determine differences between multiple groups by performing one‐way analysis of variance (ANOVA) and using the Student–Newman–Keuls post hoc test for all pair‐wise comparisons in cases where ANOVA revealed differences between groups. Statistical significance was assumed when *P *<* *0.05.

## Results

### Synthesis and characterization of collagen‐targeted (T‐AuNP) and scrambled AuNP (S‐AuNP)

Peptide‐fluorophore‐polyethylene glycol (PEG) functionalized AuNPs were synthesized as shown in Figure 1A. Briefly, citrate‐stabilized 13 nm diameter AuNPs were synthesized and surface functionalized with either a 200‐fold molar excess of the targeted (CBP‐AF546‐PEG24‐Disulfide; T‐AuNP) or a 300‐fold molar excess of the scrambled (SCR‐AF546‐PEG24‐Disulfide; S‐AuNP) molecule (Fig. 1B), followed by addition of PEG‐SH (MW = 1000) to passivate remaining unfunctionalized nanoparticle surface area. These empirically determined ratios enabled equivalent T‐ and S‐ligand loading to the AuNP surface. Functionalization of the AuNPs did not disrupt the spherical AuNP core, measured by TEM and UV‐Vis spectroscopy (Fig. 1C and D). Additionally, a fluorescent peak for the Alexa Fluor 546 fluorophore (AF546) was observed in both the T‐AuNP and S‐AuNP, but not in the starting 13 nm AuNP, indicating successful ligand adsorption (Fig. 1D).

The T‐AuNP and S‐AuNP had similar sizes (19.9 ± 0.5 nm for the T‐AuNP vs. 17.4 ± 0.2 nm for the S‐AuNP), surface charges (−43.8 ± 0.4 mV vs. −43.7 ± 0.6 mV), and *λ*_max_ (526 nm vs. 528 nm), a marker of stability, as measured by dynamic light scattering (DLS), zeta potential, and UV‐Vis spectroscopy, respectively (Table**** 1). The number of ligands per nanoparticle, determined by first dissolving the AuNP core with potassium cyanide and then quantifying the fluorescence from the AF546 fluorophore in each sample, did not significantly differ between the particle types (187 ± 23 ligands per T‐AuNP vs. 184 ± 19 ligands per S‐AuNP; Table 1), demonstrating equivalent loading between the T‐AuNP and S‐AuNP groups.

**Table 1:** Characterization of T‐AuNPs and S‐AuNPs for size, surface charge, stability, and ligand loading onto the AuNP

	Size (nm)	Zeta (mV)	*λ*_max_ (nm)	Ligands/AuNP
T‐AuNP	19.9 ± 0.5	−43.8 ± 0.4	526	187 ± 23
S‐AuNP	17.4 ± 0.2	−43.7 ± 0.6	528	184 ± 19

### T‐AuNP binds with specificity to vascular injury

**Figure 2:**
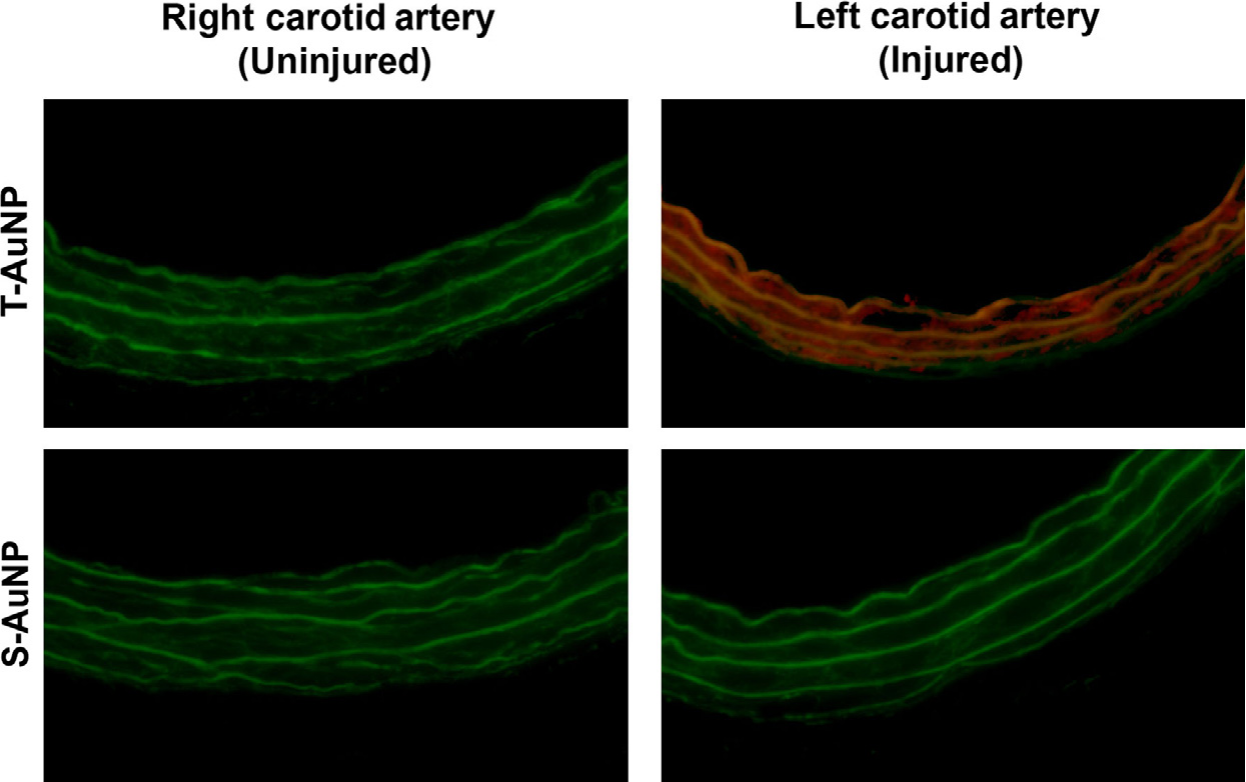
T‐AuNP binds to the site of artery injury. Representative rat carotid artery sections from uninjured (right) and injured (left) arteries injected with the targeted gold nanoparticle (T‐AuNP, *N* = 3, 2.0 mg/kg) or scrambled gold nanoparticle (S‐AuNP, *N* = 3, 2.7 mg/kg) for 20 min. Fluorescence was detected in the injured left carotid arteries of animals injected with the T‐AuNP and not in their contralateral uninjured right carotid arteries. No fluorescence was detected in either arteries with administration of the S‐AuNP. Red indicates the presence of the Alexa Fluor 546 fluorophore. Green indicates elastic lamina autofluorescence. Images obtained using 25× magnification with exposure time of 200 msec.

Following rat carotid artery balloon injury and systemic injection of the T‐AuNP (2.0 mg peptide‐fluorophore‐PEG conjugate/kg body weight, *N* = 3), fluorescence was detected 20 min later at the site of left carotid arterial injury (Fig. 2). Fluorescence was prominent circumferentially and was dispersed throughout the layers of the arterial wall. To determine whether the fluorescence was specific to the site of injury, we evaluated the fluorescent signal in the left carotid artery proximal to the site of injury, including as far proximal as the aortic arch. No T‐AuNP fluorescence was observed in these sections, which were also negative for MPO or ED1 staining, but positive to CD31 staining (data not shown). Similarly, no fluorescence was detected in the contralateral, uninjured right carotid artery. Finally, no fluorescence was detected after injection of the S‐AuNP (2.7 mg/kg, *N* = 3) at either the injured left or uninjured right carotid arteries (not shown).

**Figure 3:**
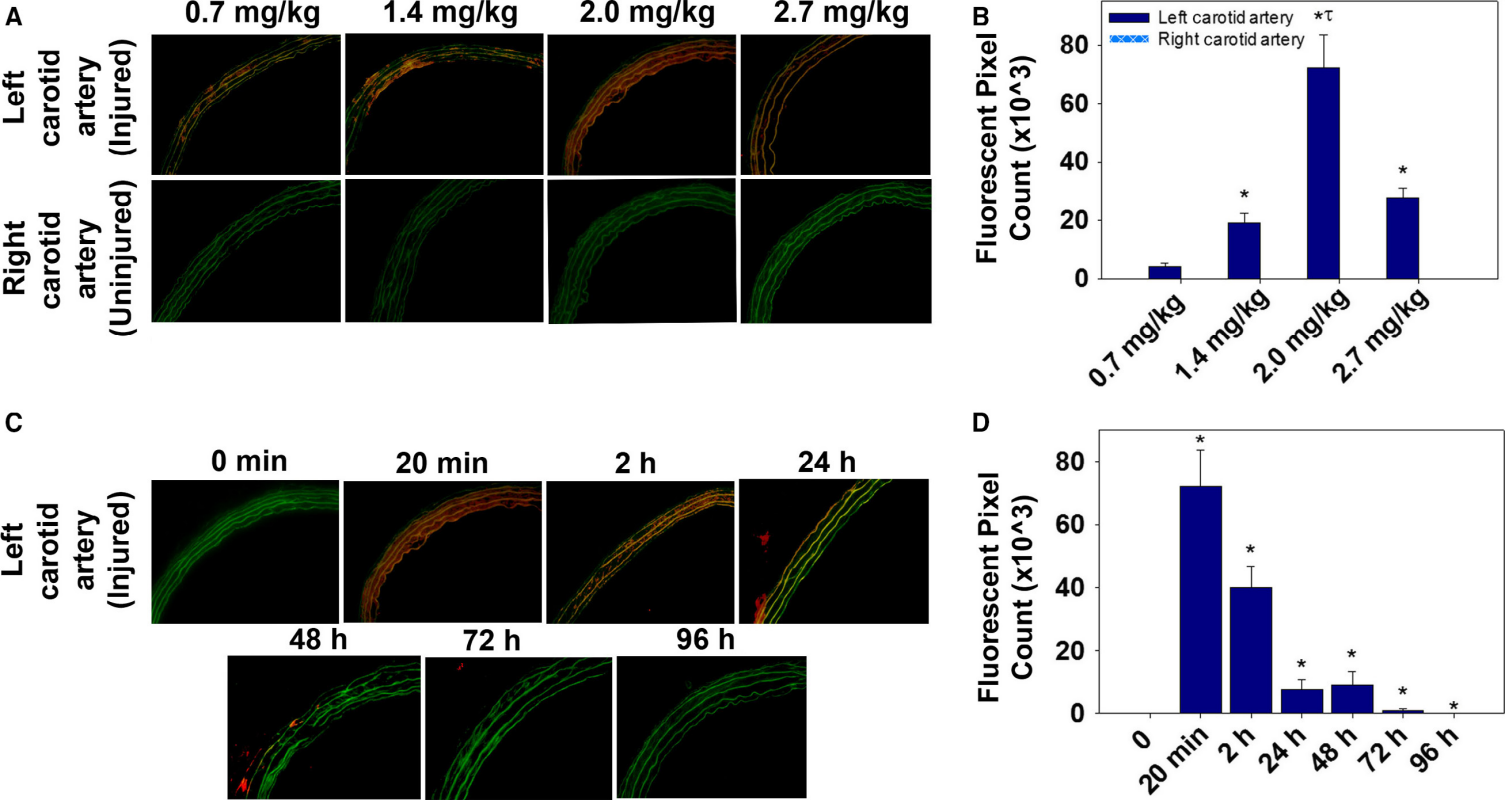
Evaluation of the optimal concentration and binding duration of the T‐AuNP. (A) Effect of T‐AuNP concentration on binding to arterial injury, as determined by fluorescent microscopy. Representative sections of injured left carotid arteries and uninjured right carotid arteries at 0.7 mg/kg (*N* = 3), 1.4 mg/kg (*N* = 5), 2.0 mg/kg (*N* = 3) and 2.7 mg/kg (*N* = 3). Red indicates presence of the Alexa Fluor 546. Green indicates elastic lamina autofluorescence. (B) Quantification of T‐AuNP fluorescence at the site of injury. The greatest T‐AuNP binding to injury occurs at a dosage of 2.0 mg/kg (*P < 0.001 vs. corresponding right carotid artery; ^t^P<.001 vs. 0.7 mg/kg, 1.4 mg/kg, and 2.7 mg/kg left carotid arteries). (C) Binding duration of the T‐AuNP (2.0 mg/kg) at the site of injury, as determined by fluorescent microcopy (*N* = 3 rats per time point), ranging from 0 min to 96 h. (D) Quantification of T‐AuNP fluorescent signal at the site of vascular injury over time. *P < 0.05. Images obtained using 25× magnification with exposure time of 200 msec.

A dose escalation study was conducted to determine the concentration of T‐AuNP required for optimal fluorescence, and thereby binding, to arterial injury. After carotid arterial injury and systemic injection of the T‐AuNP for 20 min, fluorescence was consistently detected at the site of left carotid artery injury at all time points tested, but was negligible at the contralateral uninjured right carotid artery (Fig. 3A and B, *P* < 0.001). A dosage of 2.0 mg/kg (*N* = 3) consistently provided the greatest fluorescence at the site of left carotid arterial injury (Fig. 3A). This fluorescent signal was statistically superior to that of 0.7 mg/kg (*N* = 3), 1.4 mg/kg (*N* = 5), and 2.7 mg/kg (*N* = 3) (Fig. 3B, *P* < 0.001). No fluorescence was detected in either the injured left carotid artery or the uninjured right carotid artery after systemic injection of the S‐AuNP at both 1.4 mg/kg (*N* = 4) and 2.7 mg/kg (*N* = 3) (not shown).

### T‐AuNP remains bound to arterial injury for up to 96 h

We then determined the duration of binding of the T‐AuNP to the site of injury. As shown in Figure 3C and D, the most prominent fluorescent signal detected at the site of arterial injury is evident 20 min after systemic injection of the T‐AuNP (2.0 mg/kg, *N* = 3, *P* < 0.05 vs. 0 min). Fluorescence at the site of arterial injury diminishes as the duration of circulation time increases, though remains statistically significantly greater than noninjected control animals throughout the study period (*P* < 0.05). The pattern of fluorescence shifts from binding within the arterial media and toward adventitial binding as the duration of circulation time increases. By 96 h (*N* = 3), scant fluorescence is detected in the injured artery or adventitia.

### T‐AuNP biodistribution as a function of time

**Figure 4:**
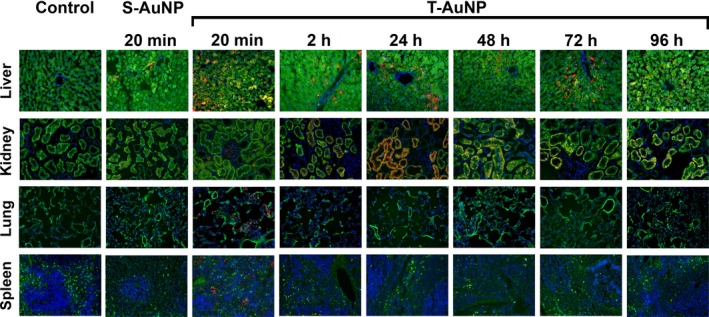
Organ localization of the T‐AuNP and S‐AuNP after systemic injection. Organ localization of the T‐AuNP (2.0 mg/kg, *N* = 3 rats/time point) was evaluated over 96 h and compared to noninjected control organs and to S‐AuNP injected organs (2.7 mg/kg, *N* = 3 rats) after 20 min of circulation. Red indicates the presence of the Alexa Fluor 546 fluorophore. Green indicates elastic lamina autofluorescence. Images obtained using 25× magnification with exposure time specific for each organ (liver 200 msec, kidney 200 msec, lung 6 msec, spleen 28 msec).

Next, we assessed fluorescence in organs (liver, kidney, lung, and spleen) at different time points following systemic injection of the T‐AuNP (2.0 mg/kg, *N* = 18, Fig. 4). Within the liver, fluorescence is evident throughout the entire 96‐h circulation time, with a prominence of fluorescence in a perisinusoidal pattern radiating from the central vein. Within the kidney, fluorescence is first prominent in the glomerulus at the 20‐min time point, then transitions to fluorescence within the nephron itself by 2 h, and with a peak fluorescence observed at 24 h. After 24 h, fluorescence diminishes in the nephron up to 96 h. Within the lung, fluorescence is noted at the 20‐min time point, with no further fluorescence detected at any other time points. Likewise, within the spleen, a small amount of fluorescence is noted at the 20‐min time point, with no fluorescence observed at later time points. Fluorescence was additionally assessed 20 min after injection of the S‐AuNP (2.7 mg/kg, *N* = 3). A similar pattern of fluorescence as with the T‐AuNP was evident in the liver with injection of the S‐AuNP. A scant amount of fluorescence was observed in the kidney nephron, and no fluorescence was observed in the lung or spleen. Overall, these data suggest that the nanoparticle conjugate, or at minimum the molecular fluorophore, is cleared from the bloodstream by both the kidney and liver.

### T‐AuNP does not induce inflammation or architectural changes in arteries or organs

**Figure 5:**
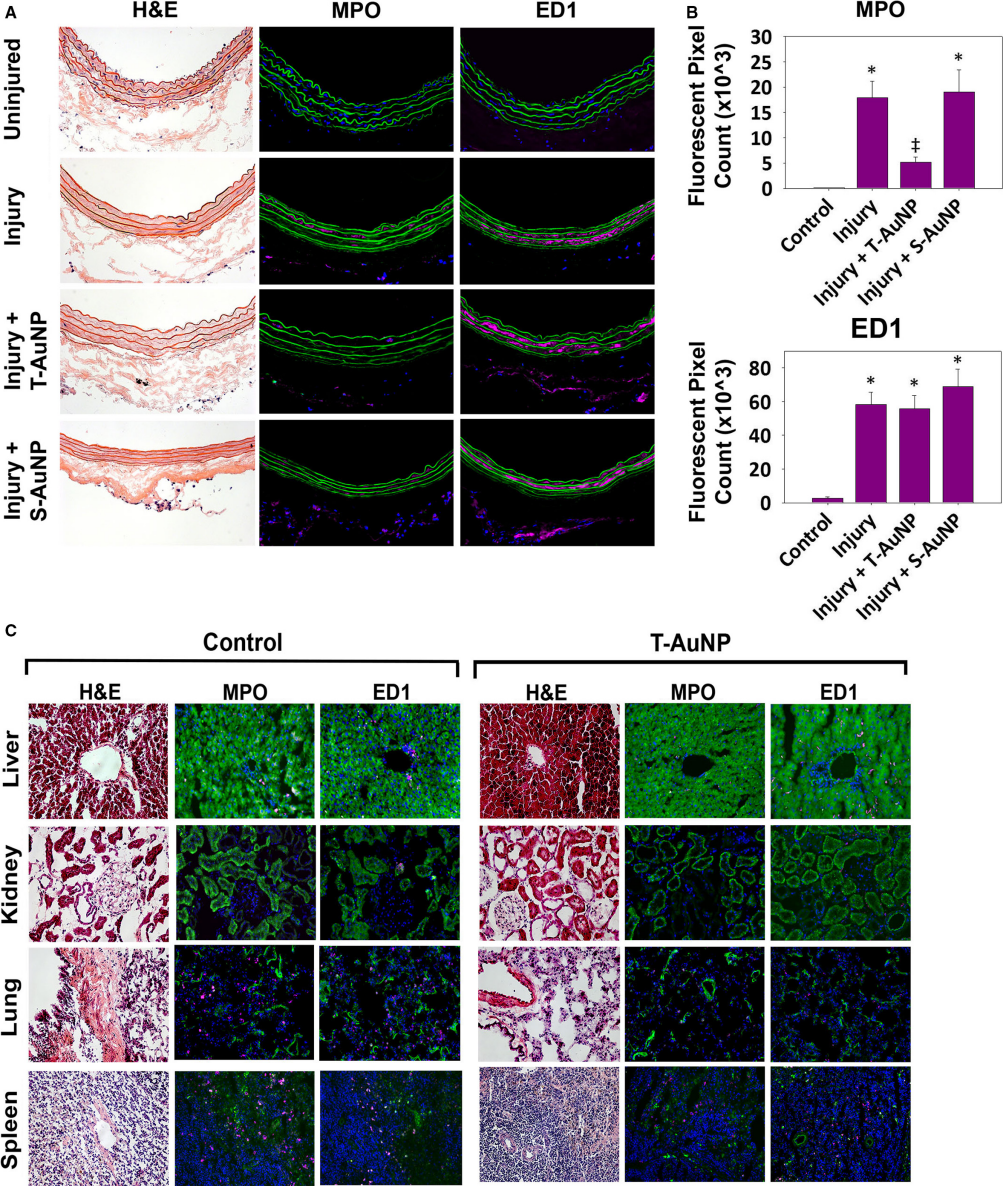
T‐AuNP does not induce neutrophil or monocyte/macrophage infiltration into the organs. (A) Representative artery sections from rats injected with the T‐AuNP (2.0 mg/kg, *N* = 3) or the S‐AuNP (2.0 mg/kg, *N* = 3) for 24 h, compared to noninjured control right carotid arteries (*N* = 3) and injury alone left carotid arteries (*N* = 3). (B) Quantification of myeloperoxidase (MPO) and ED1 immunofluorescent stains for neutrophils and monocytes/macrophages, respectively. *P < 0.001 versus control; ^‡^ *P* = 0.002 versus injury alone and versus injury + S‐AuNP. (C) Representative organ sections at 24 h from rats injected with the T‐AuNP (2.0 mg/kg) compared to noninjected control organs. Hematoxylin–eosin (H&E) stain for architecture, MPO immunofluorescent stain for neutrophils, and ED1 immunofluorescent stain for monocytes/macrophages (*N* = 3 rats). Green indicates autofluorescence. Magenta indicates neutrophil or monocyte/macrophage presence. Images obtained using 25× magnification with exposure times specific for each organ and antibody (MPO: arteries 300 msec, organs 700 msec; ED1: arteries 250 msec, organs 160 msec).

24 h after arterial injury and systemic injection of either the T‐AuNP or S‐AuNP, neutrophil (MPO) and monocyte/macrophage (ED1) infiltration was assessed in the arteries and organs. As shown in Figure 5A and B, there was undetectable neutrophil or monocyte/macrophage infiltration in the contralateral uninjured right control arteries (*N* = 3). There was a statistically significant increase in neutrophils in the injury alone (*N* = 3) and injury with S‐AuNP injected animals (*N* = 3) (*P* < 0.001). There was no significant difference in neutrophil infiltration between the uninjured control arteries and the injured with T‐AuNP arteries (*N* = 3) at 24 h (*P* = 0.199). Additionally, there was no significant difference in the level of monocyte/macrophage infiltration between the injury alone (*N* = 3), injury with T‐AuNP (*N* = 3), and injury with S‐AuNP arteries (*N* = 3) at 24 h (*P* = 0.829). Compared to noninjected control organs, there was no difference in neutrophil or monocyte/macrophage infiltration in the liver, kidney, lung, and spleen (Fig. 5C). H&E staining demonstrates no architectural changes in the liver, kidney, lung, or spleen with injection of the T‐AuNP (Fig. 5C).

### T‐AuNP binds to and are taken up by endothelial cells, smooth muscle cells, and fibroblasts in vitro

**Figure 6:**
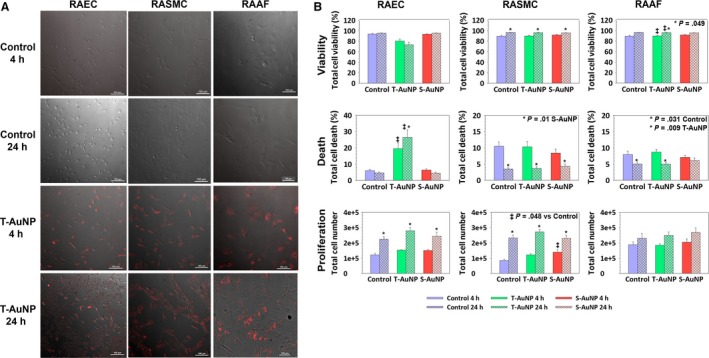
Impact of T‐AuNP and S‐AuNP treatment on vascular cells in vitro and on endothelial cells in vivo. (A) Binding of the T‐AuNP (45 nmol/L) to RAEC, rat aortic smooth muscle cells (RASMC), and RAAF in vitro at 4 h and 24 h versus control cells treated only with complete media. Red indicates the presence of the Alexa Fluor 546 fluorophore. *N* = 2 experimental replicates. (B) Trypan blue analysis of RAEC, RASMC, and RAAF after 4 h and 24 h of exposure to the T‐AuNP (45 nmol/L) and S‐AuNP (45 nmol/L). *N* = 4 experimental replicates. *P < 0.001 for 24 h versus 4 h and ^‡^ *P* < 0.001 versus control, unless otherwise noted in the graphs. Solid bars represent 4 h, hashed bars represent 24 h. RAEC, Rat aortic endothelial cells; RAAF, rat aortic adventitial fibroblasts.

Given that fluorescence was evident throughout the arterial wall following systemic injection of T‐AuNP and not just localized at the endothelial cell monolayer, we wanted to assess the ability of the T‐AuNP to bind to and become internalized within rat aortic endothelial cells (RAEC), rat aortic smooth muscle cells (RASMC), and rat adventitial arterial fibroblasts (RAAF) in vitro. Cells were exposed to 45 nmol/L T‐AuNP or S‐AuNP for 4 h or 24 h, a concentration that corresponds to systemic injection of 2.0 mg/kg in vivo (*N* = 2 replicates). As shown in Figure 6A, the T‐AuNP binds to RAEC, RASMC, and RAAF at 4 h. Fluorescent signal intensity increased over time and the pattern of fluorescence appeared perinuclear, suggesting internalization of the T‐AuNP at 24 h. However, after both 4 and 24 h of exposure to the T‐AuNP, cellular debris was noted within the RAEC wells.

### T‐AuNP does not effect cell viability, death, or proliferation in RASMC and RAAF, but induces death in RAEC in vitro

Due to the debris noted after exposure of the RAEC to the T‐AuNP, we assessed the impact of the T‐AuNP and S‐AuNP (45 nmol/L) on cellular viability, death, and proliferation in vitro (Fig. 6B). We found that the T‐AuNP and S‐AuNP had no effect on RASMC or RAAF viability, death, or proliferation after 4 h and 24 h of exposure (*N* = 4 replicates), as measured by trypan blue staining and manual cell counting, respectively. However, the T‐AuNP induced 19.5% and 26.4% cell death in RAEC at 4 h and 24 h, respectively, versus 6.0% and 4.6%, respectively, in RAEC exposed to media alone (*P* < 0.001). A corresponding decrease in percent cell viability was also noted in RAEC exposed to T‐AuNP compared to control treated RAEC, but this did not reach statistical significance (*P* = 0.496). Interestingly, the S‐AuNP had no effect on cell death or viability in RAEC and neither particle impacted RAEC proliferation.

### T‐AuNP does not impact the integrity of endothelial cells in vivo

**Figure 7:**
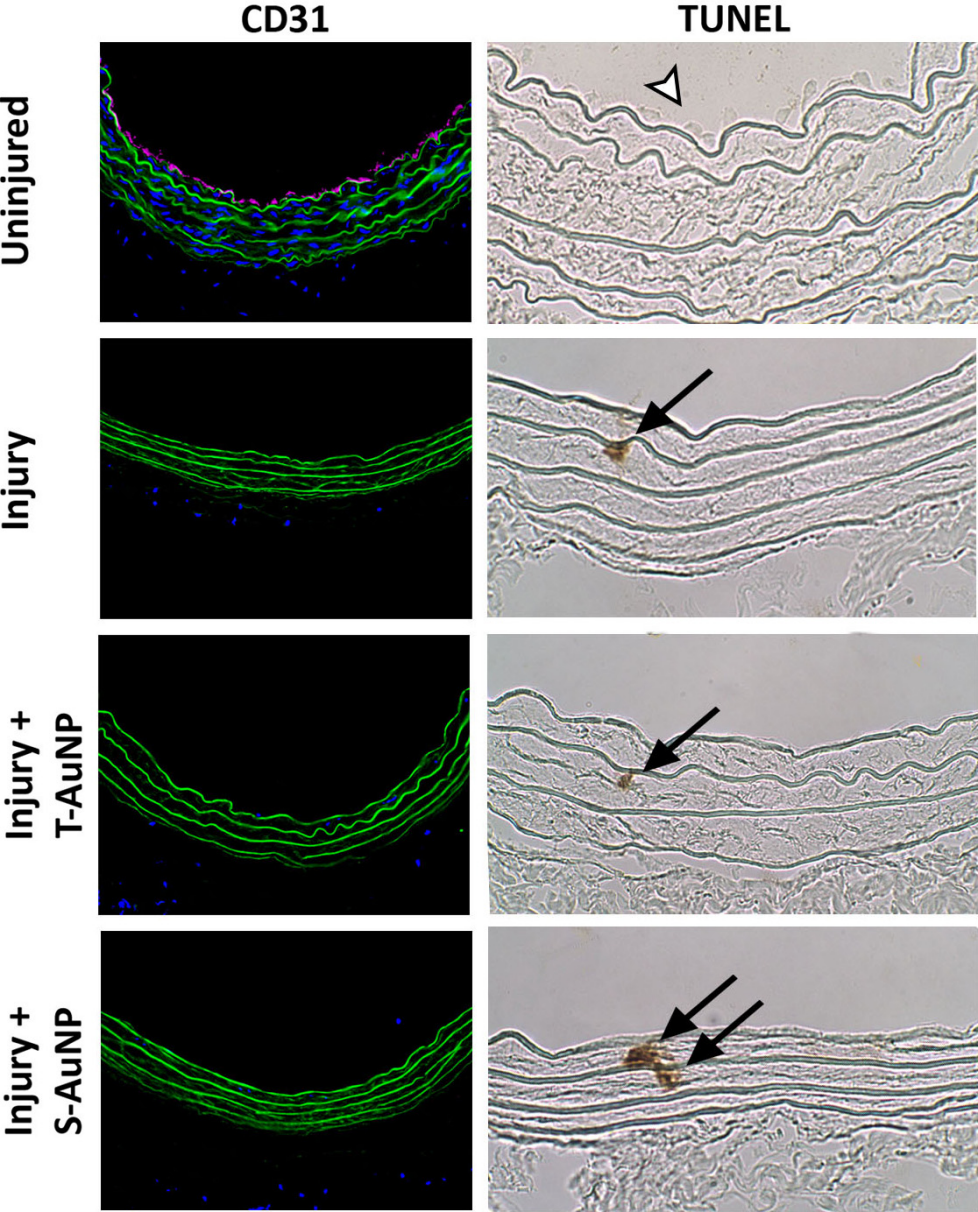
Impact of T‐AuNPs on endothelial cells in vivo. Immunofluorescent staining for endothelial cells using an anti‐CD 31 antibody revealed an intact endothelial cell monolayer in the uninjured control carotid artery (*N* = 3). As expected post balloon angioplasty, there is no endothelial cell monolayer of the injured left carotid arteries in the injury alone, injury + T‐AuNP, and injury + S‐AuNP treatment conditions. Green indicates autofluorescence of the elastic lamina. Magenta indicates endothelial cells. Images obtained using 25× magnification with exposure time of 400 msec. TUNEL staining for apoptosis revealed no evidence of endothelial cell apoptosis in the uninjured control carotid arteries, with a prominent endothelial cell monolayer (white arrowhead). Mild apoptosis was noted in the media of balloon‐injured left carotid arteries, as expected. Black arrows indicate apoptotic cells. Representative images obtained using 40× magnification.

Since the T‐AuNP was noted to induce RAEC death in vitro, we assessed the integrity of the endothelial cell monolayer in the contralateral uninjured right carotid artery and ipsilateral left carotid artery proximal to the site of injury in vivo after systemic injection of the T‐AuNP (2.0 mg/kg) for 24 h. Immunofluorescent staining for CD31 revealed an intact endothelial cell monolayer present at the luminal surface of the uninjured right carotid artery (Fig. 7) and the left carotid artery proximal to the site of injury (not shown). Immunofluorescent staining of the injury alone, injury with T‐AuNP injection, and injury with S‐AuNP injection revealed the characteristic absence of an endothelial cell monolayer which occurs following balloon angioplasty and monolayer denudation. TUNEL staining demonstrated no endothelial apoptotic cell death in the uninjured right carotid artery and evidence of an intact endothelial cell monolayer, suggesting that the T‐AuNP induced RAEC cell death may have been an artifact of the in vitro cell culture methodology. There was mild apoptosis in the media of the injured left carotid arteries, as is expected following balloon angioplasty (Fig. 7).

### T‐AuNP induces minimal and transient changes in the blood chemistries

Blood was collected at the time of organ harvesting and analyzed for hepatic, renal, and metabolic alterations (Table 2). The only statistically significant blood chemistry abnormality was an elevation of the potassium level at all four time points (*P* = 0.004). There was a transient elevation of the aspartate aminotransferase (AST) level at day 1 (*P* = 0.034) and of the cholesterol level at day 3 (*P* = 0.006), both of which returned to baseline by day 4.

**Table 2:** Hepatic, renal, and metabolic values of animals injected with T‐AuNP (2.0 mg/kg) over the 96‐h study period

	Control group^a^		T‐AuNP Group							
			Day 1		Day 2		Day 3		Day 4	
	Mean	SEM	Mean	SEM	Mean	SEM	Mean	SEM	Mean	SEM
Alkaline Phosphatase (U/L)	185	±19	143	±15	133	±16	143	±15	105	±18
SGPT (ALT) (U/L)	94	±13	90	±21	60	±3	50	±6	35^c^	±6
SGOT (AST) (U/L)	145	±27	379^c^	±134	162	±18	210	±64	83	±10
Creatine Phosphokinase (U/L)	565	±118	581	±412	538	±246	1035	±365	410	±54
Albumin (g/dL)	2.9	±0.1	2.8	±0.1	2.7	±0.1	2.8	±0.1	2.7	±0.1
Total Protein (g/dL)	5.5	±0.1	6	±0.4	5.9	±0.2	6.3	±0.4	5.6	±0.1
Globulin (g/dL)	2.6	±0.1	3.2	±0.3	3.2	±0.1	3.5^c^	±0.4	2.9	±0.03
Total Bilirubin (mg/dL)			0.2	±0.0	0.1	±0.0	0.2	±0.1	0.1	±0.0
Direct Bilirubin (mg/dL)			0	±0.0	0.1	±0.1	0.03	±0.03	0.03	±0.03
Indirect Bilirubin (mg/dL)			0.2	±0.0	0.1	±0.1	0.1	±0.03	0.1	±0.03
BUN (mg/dL)	18	±1	32	±12	21	±3	39	±11	23	±1
Creatinine (mg/dL)	0.38	±0.02	0.4	±0.1	0.4	±0.1	0.5	±0.1	0.4	±0.0
Cholesterol (mg/dL)	87	±5	98	±13	114	±15	137^c^	±7	93	±11
Glucose (mg/dL)	259	±17	218	±6	189	±7	190	±5	205	±10
Calcium (mg/dL)	5–13^b^		10	±0.3	10	±0.3	9.8	±0.1	9.7	±0.03
Phosphorus (mg/dL)	7.3	±0.2	7.8	±1.1	7.3	±0.7	6.6	±0.5	7.3	±0.4
Bicarbonate (mmol/L)	26	±1	31	±5	27	±2	25	±2	28	±2
Chloride (mmol/L)			94	±6	99	±1	100	±1	99	±2
Potassium (mmol/L)	4.6	±0.1	6.2^a^	±0.3	6^a^	±0.1	5.9^a^	±0.5	5.5^a^	±0.2
Sodium (mmol/L)	142	±0.4	141	±0.0	140	±1	141.3	±0.7	140.3	±0.7

a Control Group values taken from (Bahnson et al. 2016; Rink et al. 2013) *N* = 3–6 rats.

b Reference range per IDEXX BioResearch Laboratories VetConnect.

c *P* < 0.05 with respect to the control group. *N* = 2–3 rats per T‐AuNP group.

## Discussion

We have synthesized and characterized a nanoparticle conjugate that can be systemically administered and which targets, with specificity, the site of vascular injury. The T‐AuNP remains bound to arterial injury for a minimum of 24 h, and potentially up to 96 h, and is biocompatible in vivo, inducing no inflammatory changes, no organ architectural changes, and minimal blood chemistry alterations. This research provides the foundation for further optimization of this nanoparticle conjugate for ultimate delivery of small molecule therapeutics and/or therapeutic nucleic acids to treat vascular disease in a targeted fashion.

The gold nanoparticle core of the T‐AuNP evaluated in this study affords numerous advantages in the field of targeted nanotherapeutics. Gold nanoparticles are inert (Daniel and Astruc 2004; Love et al. 2005), biocompatible constructs (Bhattacharya and Mukherjee 2008; Connor et al. 2005; Mukherjee et al. 2007; Shukla et al. 2005) that are relatively easy to synthesize with controllable size, shape, and surface charge (Niemeyer and Ceyhan 2001; Shan 2004), and are easy to characterize due to their characteristic surface plasmon resonance (Bhattacharya and Mukherjee 2008). Spherical gold nanoparticles are readily internalized within cells, as shown with our data as well as in numerous published studies (Bhattacharya and Mukherjee 2008; Connor et al. 2005; Mukherjee et al. 2007; Shukla et al. 2005; Tkachenko et al. 2003, 2004), thus providing an advantageous avenue for therapeutics delivery within cells. Moreover, AuNPs interact strongly with thiols and moderately strong with amines, thus enabling facile surface functionalization of the AuNP core.(Bhattacharya and Mukherjee 2008; Love et al. 2005). Therefore, AuNPs are an ideal scaffold for the development of nanoconstructs for drug delivery to treat diseases across numerous medical and surgical specialties.

The T‐AuNP conjugate evaluated in this study utilizes the strong gold‐sulfur interaction to surface functionalize the AuNP core with a collagen‐binding peptide sequence for targeting the AuNP to exposed collagen IV at the site of injury. The collagen‐binding peptide sequence used in this study is the same peptide discovered by Chan et al. (2010)in and used in other studies from our laboratory to target vascular injury (Bahnson et al. 2016; Chan et al. 2011, 2010; Moyer et al. 2015). The T‐AuNP and S‐AuNP syntheses were optimized to produce nanoparticles with maximal loading of the targeted and scrambled peptides, in order to demonstrate the proof‐of‐concept that a collagen‐targeted spherical gold nanoparticle can localize to the site of vascular injury following systemic administration. It is possible that a reduced number of targeting peptides per nanoparticle will produce similar results, providing additional space for the loading of therapeutics.

Systemically injected and targeted nanotherapeutics do exist outside of the primary vascular arena. Recognition of the importance of pathophysiologically leaky vasculature in injury has led to a variety of systemically injectable nanotherapeutic innovations for treating inflammation and cancer (Cabral et al. 2015; Christie et al. 2012; Guan et al. 2014; Jiang et al. 2014; Lobatto et al. 2015; Morgan et al. 2016; Rink et al. 2013; Shenoi et al. 2013). However, no such systemically injected nanoconstruct exists in the clinical vascular arena to target and repair the site of arterial injury. Chan et al. (2011) developed a lipid‐polymeric nanoparticle surface functionalized with the collagen‐binding peptide and the cancer chemotherapeutic agent paclitaxel, and demonstrated reduced neointimal hyperplasia at 2 weeks. However, as an antiproliferative agent, paclitaxel inhibits all cellular proliferation, has no vascular reparative benefits, and has been implicated in increased rates of in‐stent thrombosis versus bare‐metal stents (Palmerini et al. 2012). The T‐AuNP developed in here is unique in the ability to surface functionalize the AuNP core and/or modify the collagen‐binding peptide‐molecular fluorophore‐PEG conjugate with a variety of moieties, from small molecule drugs such as everolimus and sirolimus, to therapeutic nucleic acids such as short‐interfering RNA and antisense DNA sequences, to contrast agents such as gadolinium. Thus, it is easy to envision the development of a T‐AuNP construct containing a combination of small molecule drugs and imaging agents to prevent neointimal hyperplasia.

We found that the T‐AuNP bound to the site of arterial injury for at minimum 24 h, consistent with binding of the collagen‐targeted lipid nanoparticle described previously (Chan et al. 2011). This duration of binding to exposed collagen has important clinical implications for targeted delivery of therapeutics to the site of injury. Based upon our previously published work, the duration of binding of the T‐AuNP appears to be sufficient to have a durable inhibition of neointimal hyperplasia (Bahnson et al. 2016). Additionally, a benefit of this construct is that repeat intravenous dosing of the T‐AuNPs could extend this therapeutic window, which should be sufficient to have a beneficial clinical impact on vascular repair following arterial injury.

Gold nanoparticles have been reported to be biocompatible in vitro and in vivo (Connor et al. 2005; Hainfeld et al. 2006; Mukherjee et al. 2007; Shukla et al. 2005). While we found that systemic injection of the T‐AuNP was biocompatible in vivo with no induction of inflammation, no major blood chemistry alterations, and an intact endothelial cell monolayer, we did find that exposure of RAEC to the T‐AuNP did induce cell death in vitro. Interestingly, the S‐AuNP did not induce similar endothelial cell death and the T‐AuNP did not alter viability, death, or cellular proliferation of VSMC. It is possible that the T‐AuNPs may be acting similar to cell penetrating peptides in vitro, or that the T‐AuNPs disrupted adhesion of the RAECs to the culture dish, leading to apoptosis. The reason we did not observe changes with the endothelial cell monolayer in vivo is likely because the in vitro environment only vaguely recapitulates the in vivo environment. Cell culture is a static environment, constantly exposing the cells to the nanoparticle, whereas injection of the T‐AuNP in the blood stream is a dynamic process in which the nanoparticles are never stagnant. However, it is important to note that the finding of endothelial cell death upon direct exposure to the T‐AuNP delivery vehicle may be inconsequential, given that the agent binds to exposed collagen at sites of endothelial denudation and no in vivo endothelial cell apoptosis was observed.

Systemic administration of the T‐AuNP did result in fluorescent signal in organs, with more fluorescence noted in the liver and kidney, and only scant fluorescence in the lung and spleen at the earliest time points. These data suggest that the T‐AuNP, or at the least the molecular fluorophore, may be cleared from the bloodstream by both the liver and kidney. However, since we are detecting fluorescence, we are unable to definitively state if the fluorescence is due to the presence of the T‐AuNP or due to the fluorescent tag cleaved from the AuNP upon degradation. Further study of the biodistribution and pharmacokinetics of the T‐AuNP will elucidate the specific pattern and time frame of nanoparticle clearance. Blood chemistry analysis demonstrated elevations in potassium levels throughout all collection time points. Sample collection data reported from the laboratory did note mild hemolysis of the samples, which likely accounts for the observed potassium elevations. However, we cannot rule out decreased renal excretion as a potential cause of the hyperkalemia. Further analysis in a larger follow‐up study is warranted.

Based on the in vitro and in vivo data, the T‐AuNPs appear to be internalized by all three major cell types; however, the mechanism by which T‐AuNPs become internalized was not investigated. While other studies have demonstrated AuNP cellular internalization,(Bhattacharya and Mukherjee 2008; Connor et al. 2005; Mukherjee et al. 2007; Shukla et al. 2005; Tkachenko et al. 2003, 2004) a major determinant of cellular internalization is how the AuNP is surface functionalized. The specific cellular internalization mechanism for the T‐AuNP should be further evaluated.

In conclusion, we have demonstrated through this initial proof‐of‐concept study that a spherical, biocompatible and systemically administered nanoparticle conjugate can be targeted specifically to the site of vascular injury. This nanoparticle construct provides a platform for further optimization and drug loading for targeted delivery of therapeutics to treat vascular disease.

## Conflict of Interest

None declared.
